# Application of remote sensing to identify Copper–Lead–Zinc deposits in the Heiqia area of the West Kunlun Mountains, Chinas

**DOI:** 10.1038/s41598-020-68464-7

**Published:** 2020-07-23

**Authors:** Yu-Hai Fan, Hui Wang

**Affiliations:** 1 School of Earth Science and Land and Resources, Chang’an University, Xi’an, 710045 China; 2 Geological Exploration Institute of Aerial Photogrammetry and Remote Sensing Bureau, Xi’an, 710199 China

**Keywords:** Solid Earth sciences, Mineralogy

## Abstract

The harsh natural environment and inaccessibility of the West Kunlun Mountains are barriers for their investigation via field geology. Remote sensing technology has the advantage of being efficient on a macroscale and not being restricted by terrain or road conditions in sparsely vegetated areas with exposed bedrock. This work focuses on copper–lead–zinc deposits in the Heiqia area in the West Kunlun Mountains as a case study to illustrate the application of IKONOS remote sensing images as major data sources to fabricate a standard image map, the extraction of information on ore-controlling factors and mineralization through the use of image enhancement methods, and the interpretation of remote sensing data to identify mineral resources. Alteration anomaly information was extracted from ASTER data, verified via field survey and sampling, and used to develop a remote sensing model for utilization in future prospecting efforts. The results of the survey showed that in IKONOS (band 3, 2, and 1 synthesis) images, the copper mineralization zone exhibits interlaced gray-white, blue-gray, and blue tones in a narrow strip-like pattern, while the lead–zinc mineralization zone shows gray-white, light gray-yellow, and yellowish-brown tones in a strip-like pattern. The main remotely sensed alteration anomalies are characteristic of hydroxyl. Six hydroxyl anomalies were delineated in the study area, of which five were found to be copper–lead–zinc deposits. The location of the ore bodies coincides well with the ASTER anomalies extracted. Two Cu–Pb–Zn mineralization belts are present in the study area. The ore-bearing rock series of belt No. I is phyllite interbedded with metasandstone, and the ore comprises mainly copper deposits supplemented by lead–zinc deposits. Belt No. II is in limestone and consists mainly of lead–zinc deposits supplemented by copper deposits. A remote sensing geological prospecting model for structurally altered Wenquangou Group copper–lead–zinc deposits with a genesis related to hot water basins is established. This provides a basis for future prospecting for similar minerals in the West Kunlun metallogenic belt.

## Introduction

Geological deposits contain different mineral and chemical compositions from surrounding rocks, and these differences are often reflected in remote sensing images in the form of spectral anomaly information. In this regard, a series of remote sensing digital image processing becomes an effective, prospective means of acquiring the geological anomaly information related closely to ore-bearing strata, mineralized alteration zones, contact metamorphic zones, and tectonic zones^1–6^.

Located at the junction of the Paleo-Asian and Tethyan tectonic domains, West Kunlun is an important part of the Qin-Qi-Kun tectonic belt of China, and serves as an important area for studying the evolution of the Tethyan Ocean as well^7–22^. The stratum, structure, metamorphism, magmatic activity, and other metallogenic geological conditions in the area are superior, with the different types of deposits that have been discovered, along with good prospects underway^23–45^.

The West Kunlun area is rich in minerals, and a large number of new mineral deposits have been discovered there in recent years including Tashkurgan iron ore deposit, Dahongliutan rare metal deposit, Altokanashi manganese deposit in Aktao County, and Huoshangyun super large lead–zinc deposit in Hetian County. These deposits have attracted widespread attention from geologists at home and abroad^46–51^. This study uses high-resolution IKONOS remote sensing data to interpret the strata, lithology, and structure in the area to highlight the ore-controlling factors and gather mineralization information. By using ASTER data to extract spectral anomalies related to copper, lead, and zinc mineralization, two copper–lead–zinc mineralization sites are identified in the Heiqia area and are then verified by field investigation. Based on a comprehensive analysis of the remote sensing and geological information gathered for the copper–lead–zinc mineralization sites, a remote sensing geological prospecting model for copper–lead–zinc deposits is established that provides a basis for future prospecting for similar minerals in the West Kunlun metallogenic belt.

## Geologic background and sampling program

**Figure 1:**
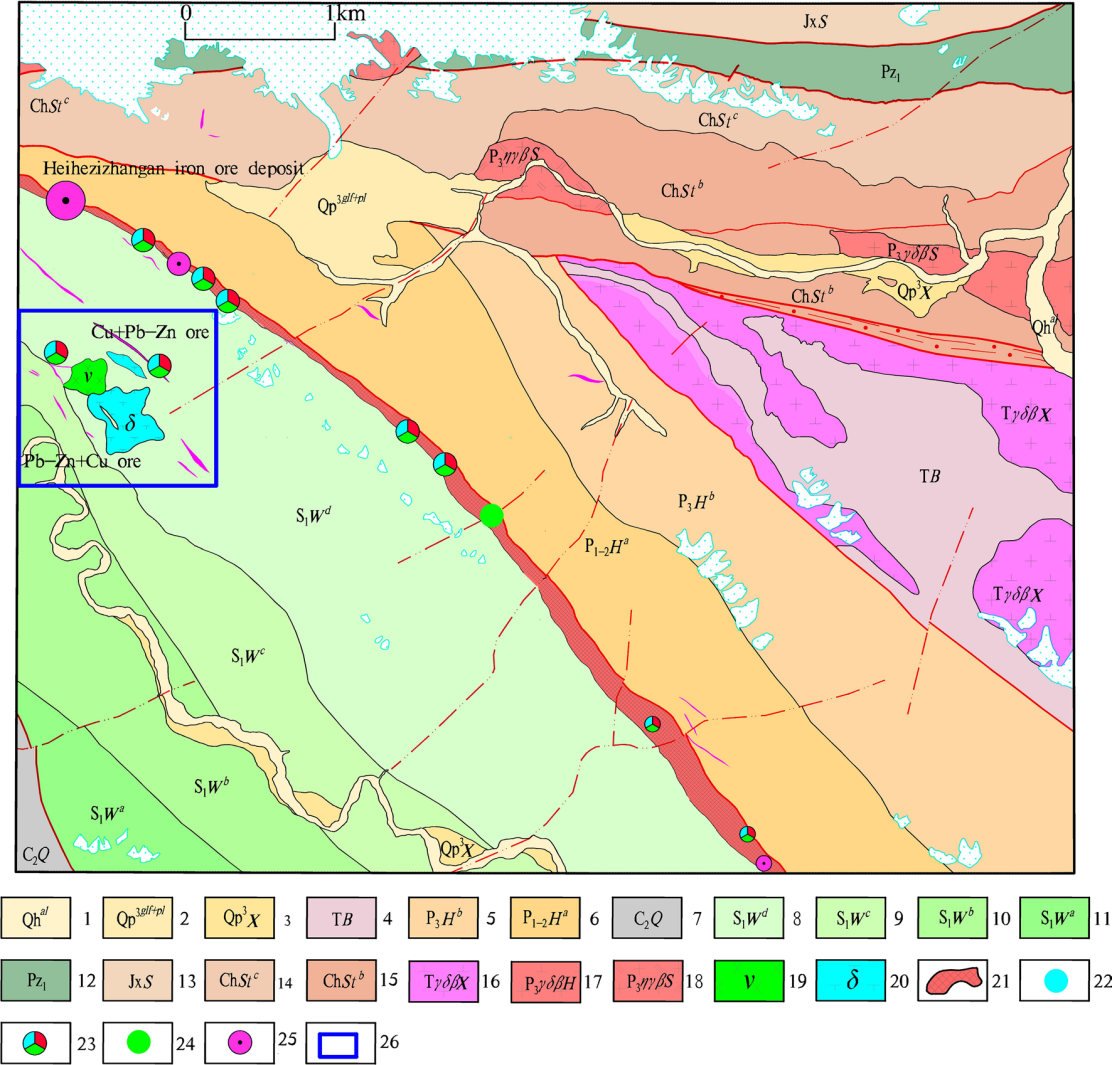
Distribution of typical ore deposits and ore (chemical) points of Heiqia polymetallic mineralization zone [(1) Alluvium of Holocene; (2) Floodplain of Late Pleistocene; (3) Xinjiang Group of Late Pleistocene; (4) Bayan Kalashan group of Triassic; (5) B Formation of Huangyangling Group in Late Permian; (6) A Formation of Huangyanling Group in Early Middle Permian; (7) Qiatier Group of Late Carboniferous; (8) D Formation of Wenquangou Group in Early Silurian; (9) C Formation of Wenquangou Group in Early Silurian; (10) B Formation of Wenquangou Group in Early Silurian; (11) A Formation of Wenquangou Group in Early Silurian; (12) Early Paleozoic Undivided; (13) Sangzhutage Group of Jixianian Period; (14) C Formation of Saitoula Group in Changcheng Period; (15) B Formation of Saitoula Group in Changcheng Period; (16) Gray granodiorite of Xieila Daoban rock mass in Triassic; (17) Gray granodiorite of Heiqia Daoban rock mass in Late Permian; (18) Light gray monzonitic granite of Saitoula rock mass in Late Permian; (19) Gabbro; (20) Diorite; (21) Iron polymetallic mineralization zone of Heiqia; (22) Lead ore point; (23) Cu–Pb–Zn ore point; (24) Copper ore point; (25) Siderite point and; (26) Research area] .This figure is generated by Yu-Hai Fan, using CorelDRAW X6 created by the CorelDRAW Team under an open license (https://www.coreldraw.com/cn/product/graphic-design-software/).

The study area span across the mountains of Kunlun and Karakorum, at the junction of the Paleo-Asian and Tethys tectonic (Kunnan-Yubei suture zone) domains, with geographical coordinates E77°15′~78°00′, N35°50′~36°30′^52–56^. Cu–Pb–Zn mineralization belt of Heiqia is located in D Formation of the Wenquangou Group in Early Silurian (S_1_*W*^d^), whereas its northeast side is a Formation of the Huangyanling Group in Early Middle Permian (P*H*^a^) along the Karatag fault boundary. On the one hand, the lithology of S_1_W^d^ is composed mainly of cinerous–gray sandy slate with argillaceous slate and metamorphic sandstone; its the top contains several layers of carbonate rocks, such as dolomite, iron dolomite, silicified dolomite, and a small amount of limestone (locally marbled) with siderite. On the other hand, the lithology of PHa is characterized by gray–black carbonaceous slate or spotted (pyrite–phenocryst) slate (Fig. 1).

## Results

### Remote sensing geological interpretation

**Figure 2:**
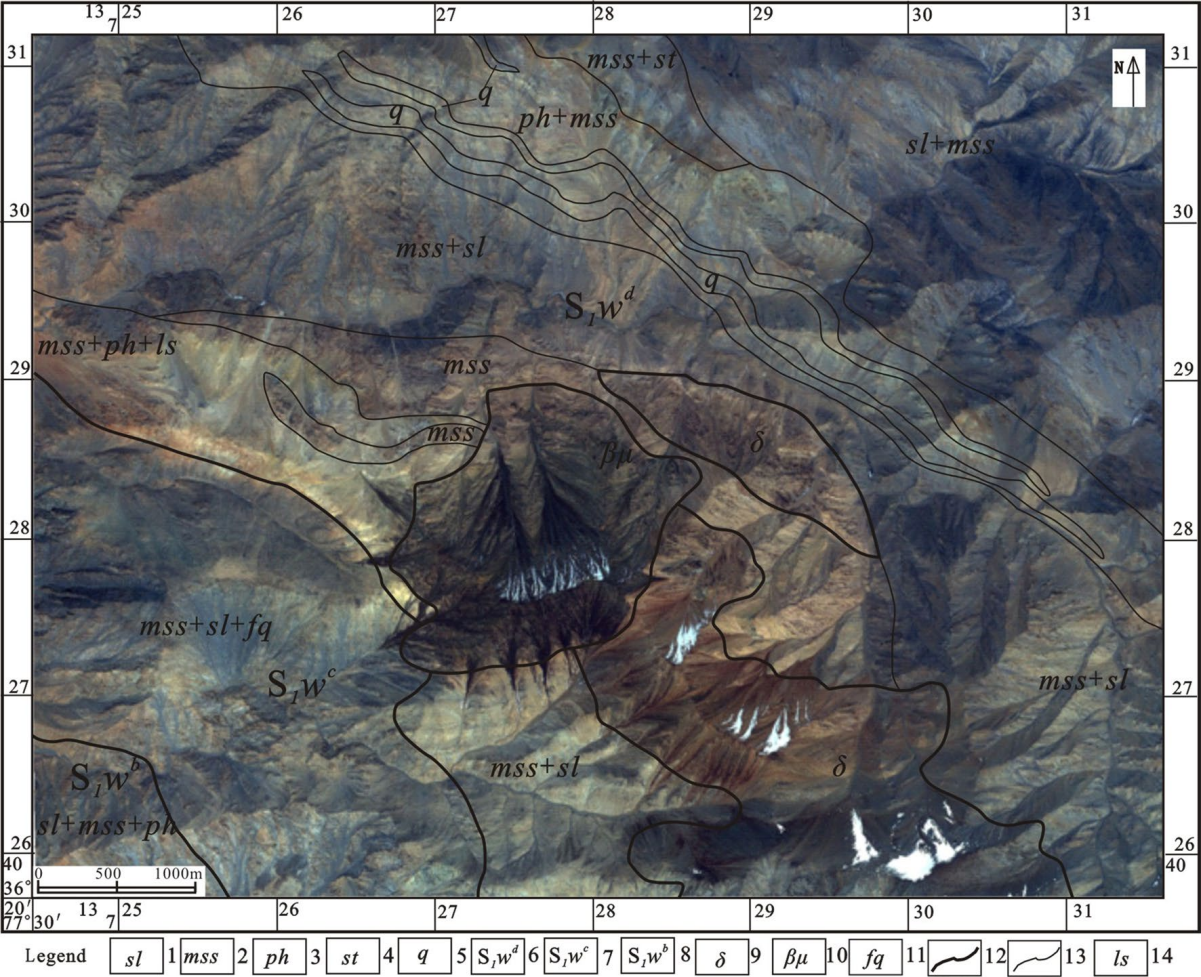
Interpreted IKONOS high-resolution remote sensing map. IKONOS was obtained from ZJ-VIEW (https://www.zj-view.com/ikonos). This figure is generated by Yu-Hai Fan, using ENVI (version 4.7) created by the ENVI Team under an open license (https://www.enviidl.com/).

Three main lithologic segments (S_1_*W*^b^, S_1_*W*^c^, S_1_*W*^d^) of the Silurian Wenquangou Group are exposed in the study area. The lithology is mainly phyllite, slate, metasandstone, feldspar–quartz sandstone, and limestone. A large number of metamorphic precipitated quartz vein belts are developed in interbedded metasandstone and phyllite. The rocks are mainly fine-grained clastic rocks. Horizontal bedding, parallel bedding, and a small number of scouring surface structures are visible in the strata, which are the general characteristics of a deep-water turbidite. A set of diabase and diorite rocks are present in the central part of the study area, intruding into Wenquangou Group metamorphic sandstone, phyllite, slate, and limestone. The intrusive rocks, the lithologic sections of the Wenquangou Group, and the quartz veins in the study area were interpreted carefully on the basis of the IKONOS high-resolution image and the 1:250,000 Kangxiwa regional geological map, and the results of the interpretation were used to construct a high-resolution remote sensing interpretation map (Fig. 2).

**Figure 3:**
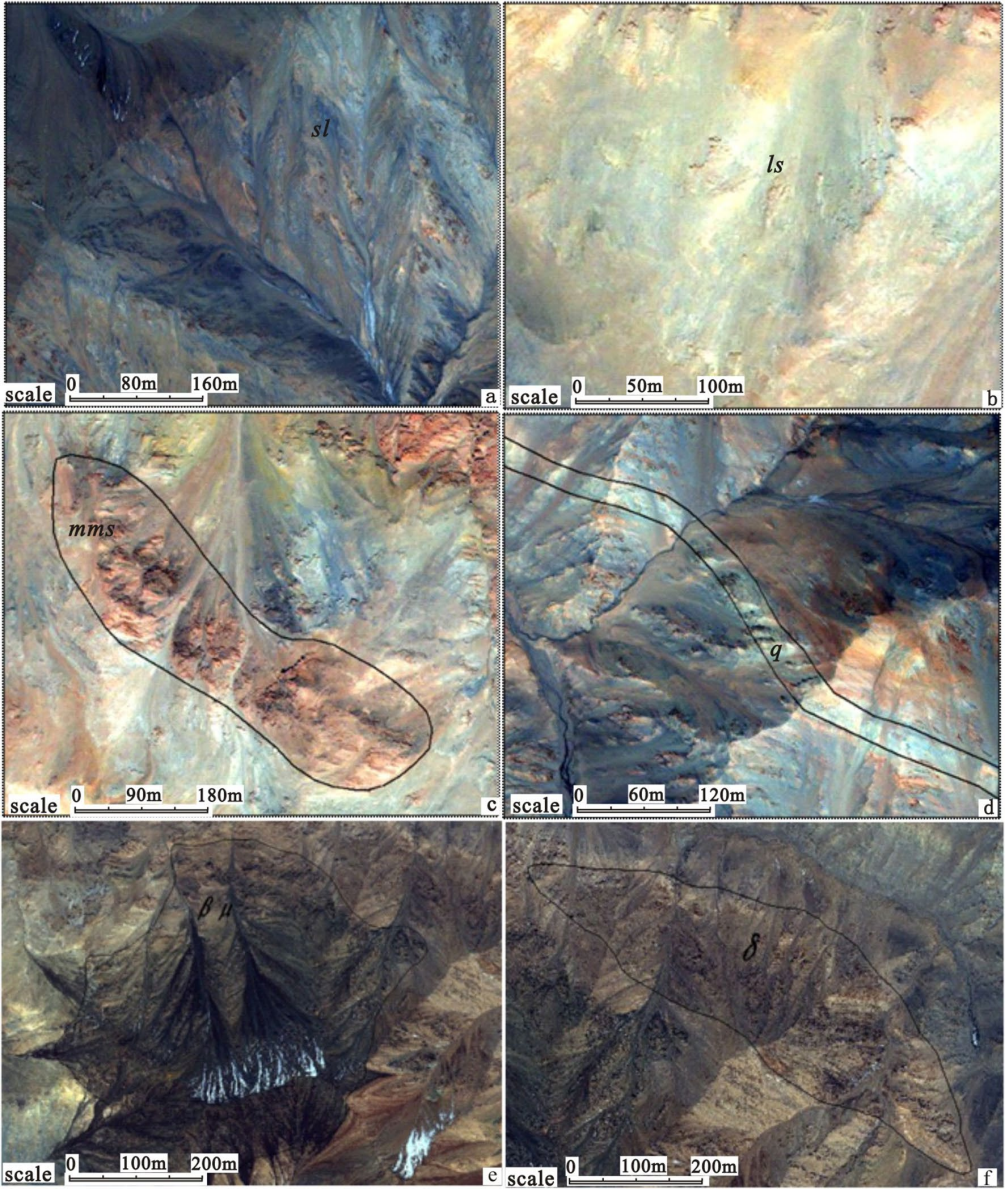
Characteristics of different lithologies in IKONOS high-resolution images. (**a**) Metamorphic precipitated quartz veins; (**b**) slate; (**c**) metasandstone; (**d**) limestone; (**e**) diabase rock; (**f**) diorite rock. IKONOS data was obtained from ZJ-VIEW (https://www.zj-view.com/ikonos). This figure is generated by Yu-Hai Fan, using ENVI (version 4.7) created by the ENVI Team under an open license (https://www.enviidl.com/).

Remote sensing data interpretation can be used to identify the following Wenquangou Group lithological assemblages. (1) Slate interbedded with metasandstone and phyllite (*sl* + *mss* + *ph*), in which the image tones are mainly blue and white, with a reddish-brown spotted texture and a layered distribution (Fig. 3a). (2) Metasandstone interbedded with slate and feldspar–quartz sandstone (*mss* + *sl* + *fq*), in which the image tones are interbedded blue and white stripes with a layered distribution. (3) Metasandstone interbedded with phyllite and limestone (*mss* + *ph* + *ls*); the image tones are light yellow, light blue, and yellow-white and show a layered distribution (Fig. 3b). (4) Metasandstone (*mss*), in which the image tone is red with a black spotted texture and a layered distribution (Fig. 3c). (5) Metasandstone interbedded with slate (*mss* + *sl*); the image tones are interbedded yellow-white and light blue, showing a striped texture. (6) Phyllite interbedded with metasandstone (ph + mss), in which interbedded light gray and light blue areas with a striped texture show a layered distribution. (7) Metasandstone interbedded with siltstone (*mss* + *st*), which has yellow and blue tones and shows a layered distribution. (8) Slate interbedded with metasandstone (*sl* + *mss*), which shows up in dark blue tones with yellow and white stripes, with a banded and speckled texture.

In this area, the quartz vein-dense zone has a white image tone and an obvious strip-like texture (Fig. 3d). Diabase rocks have a brown-black image tone and a disordered texture (Fig. 3e), whereas diorite rocks show as red-brown, also with a disordered texture (Fig. 3f).

### Extraction of information on alteration anomalies

**Figure 4:**
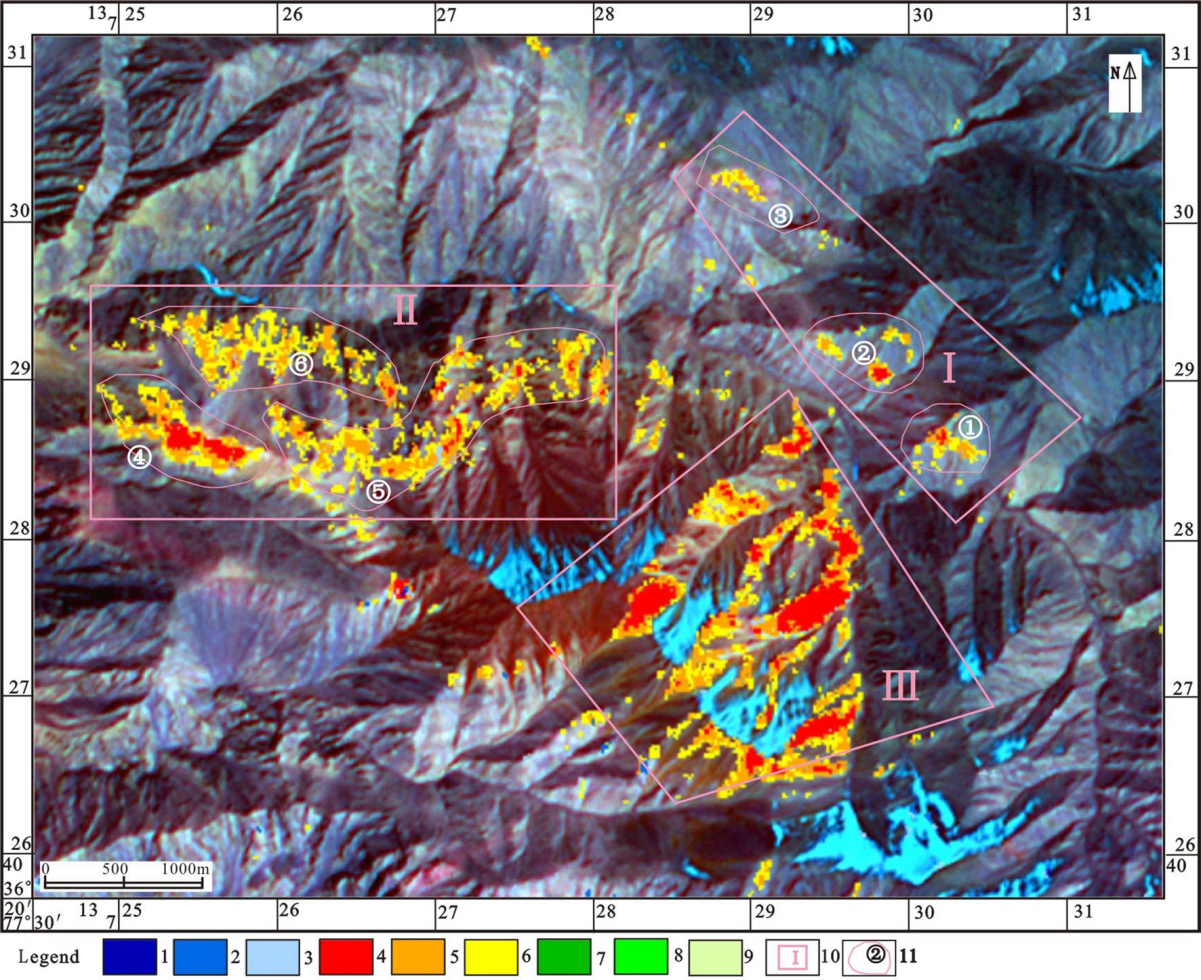
Extraction map of ASTER alteration anomalies [(1) first-order iron staining anomaly; (2) second t-order iron staining anomaly; (3) third-order iron staining anomaly; (4) first-order aluminum hydroxyl anomaly; (5) second-order aluminum hydroxyl anomaly; (6) third -order aluminum hydroxyl anomaly; (7) first-order magnesium hydroxyl anomaly; (8) second-order magnesium hydroxyl anomaly; (9) third-order magnesium hydroxyl anomaly; (10) alteration anomaly package; (11) alteration anomaly point]. ASTER data was obtained from ZJ-VIEW (https://www.zj-view.com/ikonos).This figure is generated by Yu-Hai Fan, using ENVI (version4.7) created by the ENVI Team under an open license (https://www.enviidl.com/).

According to the characteristic absorption spectra of various alteration anomalies and the corresponding relations between ASTER bands in the study area, iron stain and aluminum hydroxyl anomalies were extracted by principal component transformation. Three large-scale hydroxyl alteration anomalies were extracted from the study area by threshold selection and then anomaly separation (Fig. 4). Anomaly package No. I is located in quartz veins within phyllite interbedded with metasandstone (*ph* + *mss*) in S_1_*W*^*d*^. The ASTER remote sensing anomalies are beaded along the quartz vein band and are first- or second-order hydroxyl anomalies. Anomaly package No. II is located in metasandstone intercalated with phyllite and limestone in S_1_*W*^*d*^. The ASTER remote sensing anomaly is distributed in a cluster-band pattern and is a first-order hydroxyl anomaly. Anomaly package No. III is located in the diabase and diorite intrusions. The ASTER remote sensing anomalies are clustered and highly anomalous. Copper–lead–zinc deposits are mainly formed during mid-low temperature hydrothermal activity. Most of the ore-hosting rock series are carbonate rocks, clastic rocks, and contact zones between the rock mass and surrounding rocks. Such deposits are extremely rare in the interior of an intrusive rock mass. Therefore, anomaly package No. III was judged to be a non-mineral abnormality and was excluded.

Three ASTER hydroxyl anomaly points (①, ②, ③) (Fig. 4) can be delineated in anomaly package No. I. Among them, No.① and No.② are first-order hydroxyl anomalies, are beaded in quartz veins, and have highly anomalous values and good prospecting prospects; No. ③ is a second-order hydroxyl anomaly located in Wenquangou Group phyllite intercalated with metasandstone and has moderately anomalous values and moderate prospecting prospects.

Three ASTER hydroxyl anomaly points (④, ⑤, ⑥) (Fig. 4) can be delineated in anomaly package No. II. Among them, No.④ and No.⑥ are first-order hydroxyl anomalies that are located in the Wenquangou Group limestone. These are high-magnitude, concentrated anomalies in a cluster-belt distribution and have good prospecting prospects. Anomaly No. ⑤ is located near the contact zone of diorite and diabase with Wenquangou Group phyllite, metasandstone, and limestone. The anomaly is of high magnitude and concentrated, and it thus has prospecting significance.

## Discussion and conclusions

### Degree of spatial coincidence between ASTER alteration anomalies and field verification results

Anomaly package No. I is distributed in the east of the Yarkant River, 1 km away from National Highway 219, Keerkezijianggale team.

**Figure 5:**
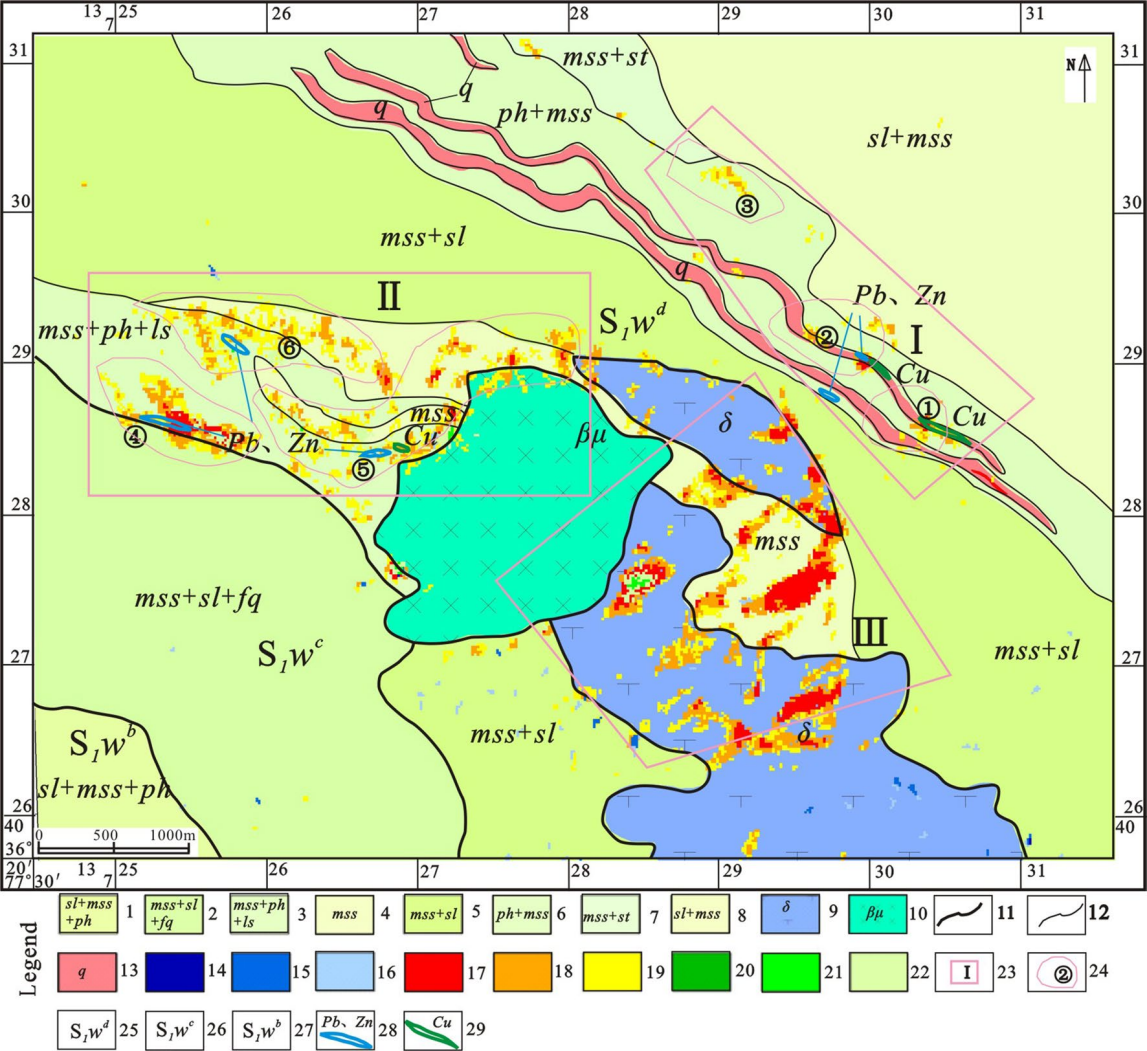
Superposition map of IKONOS high decomposition, ASTER alteration anomaly, and field validation data [(1) slate interbedded with metasandstone and phyllite; (2) metasandstone interbedded with slate and feldspar–quartz sandstone; (3) metasandstone interbedded with phyllite and limestone; (4) metasandstone; (5) metasandstone interbedded with slate; (6) phyllite interbedded with metasandstone; (7) metasandstone interbedded with siltstone; (8) slate interbedded with metasandstone; (9) diabase rocks; (10) diorite rocks; (11) stratigraphic boundary; (12) lithologic boundary ; (13) quartz vein; (14) first-order iron staining anomaly; (15) second t-order iron staining anomaly; (16) third-order iron staining anomaly; (17) first-order aluminum hydroxyl anomaly; (18) second-order aluminum hydroxyl anomaly; (19) third -order aluminum hydroxyl anomaly; (20) first-order magnesium hydroxyl anomaly; (21) second-order magnesium hydroxyl anomaly; (22) third-order magnesium hydroxyl anomaly; (23) alteration anomaly package; (24) alteration anomaly point; (25) D Formation of Wenquangou Group in Early Silurian; (26) C Formation of Wenquangou Group in Early Silurian; (27) B Formation of Wenquangou Group in Early Silurian; (28) lead–zinc orebody; (29) copper orebody]. This figure is generated by Yu-Hai Fan, using CorelDRAW X6 created by the CorelDRAW Team under an open license (https://www.coreldraw.com/cn/product/graphic-design-software/).

The three hydroxyl anomaly points (①, ②, ③) were verified. One Cu-mineralized body was found at the No. ① hydroxyl anomaly points. This has an average dew width of 4.5 m and an exposed length of about 500 m. One Cu-mineralized body and one Pb–Zn-mineralized body were found at the No. ② hydroxyl anomaly points. The average exposed width of the Cu-mineralized body was 8 m, and its exposed length was about 345 m. The average width of the Pb–Zn-mineralized body was 6 m, and its exposed length was about 150 m. No evidence of mineralization was found at the No. ③ secondary hydroxyl anomaly points, and only a small amount of silicification was observed (Fig. 5).

**Figure 6:**
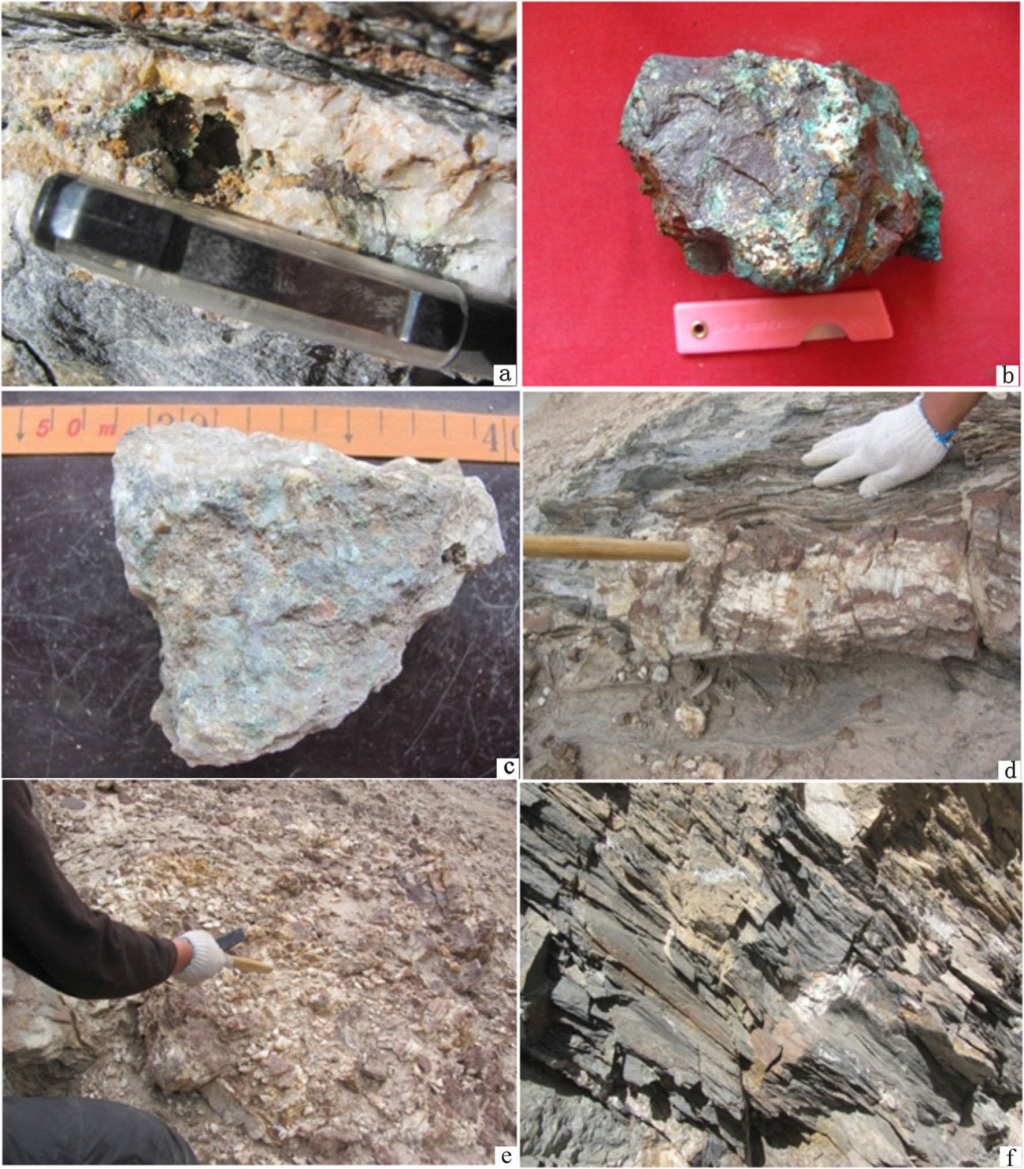
Photograph of anomaly package No. I. (**a**) Malachite in a quartz vein; (**b**) copper ore; (**c**) lead–zinc ore; (**d**) a quartz vein; (**e**) a fragmented quartz vein; (**f**) phyllite intercalated with metasandstone. These photos were taken by Yu-Hai Fan in West Kunlun in 2011.

The copper–lead–zinc ore body in anomaly package No. I occurs in quartz veins in phyllite interbedded with metasandstone, and its extension direction is parallel or nearly parallel to the strike of the strata. Its occurrence is basically the same as that of the surrounding rock (30–40° ∠ 60–70°). Ore bodies are closely related to ductile and brittle shear structural alteration zones in the quartz veins, with an outcrop width of 5–10 m and an outcrop length of 150–500 m. The surrounding rocks are sericite phyllite, sericite silty sandy slate, and fine sandstone (Fig. 6f). The alteration of the mineralization is mainly silicification and the formation of malachite and limonite (Fig. 6a). The ore minerals are chalcopyrite, pyrite, galena, and sphalerite (Fig. 6b, c). The gangue minerals are quartz and calcite (Fig. 6d, e). The ore can be classified as of quartz vein type and has a xenomorphic granular texture and a disseminated structure. The genetic type of the deposit is hydrothermal sedimentary with structural alteration.

Anomaly package No. II is distributed in the east of the Yarkant River, 200 m away from National Highway 219, Keerkezijianggale team.

The three hydroxyl anomaly points (④, ⑤, ⑥) were verified. One Pb–Zn-mineralized body was found at the No. ④ hydroxyl anomaly points, with an average width of 5 m and an exposed length of about 450 m. One Cu-mineralized body and one Pb–Zn-mineralized body were found at the No. ⑤ hydroxyl anomaly points. The average exposed width of the Cu-mineralized body was 4–5 m, and its exposed length was about 100 m. The average width of the Pb–Zn-mineralized body was 3 m, and its exposed length was about 200 m. One Pb–Zn-mineralized body was found at the No. ⑥ hydroxyl anomaly points, with an average width of 3 m and an exposed length of about 300 m (Fig. 5).

**Figure 7:**
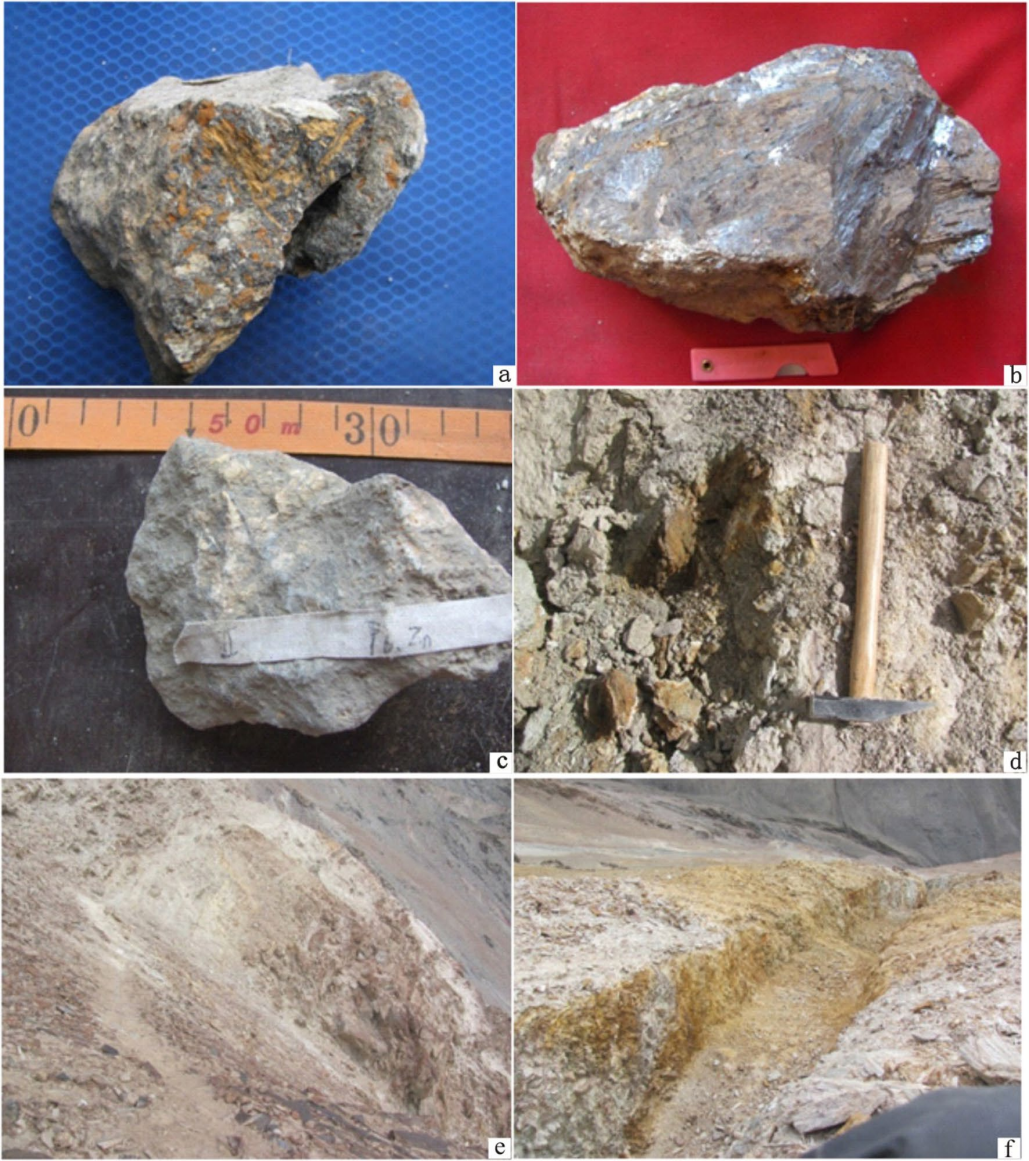
Photograph of anomaly package No. II. (**a**–**c**) lead–zinc ore; (**d**) lead–zinc ore outcrop; (**e**) ore body and surrounding rock; (**f**) trench. These photos were taken by Yu-Hai Fan in West Kunlun in 2011.

The copper–lead–zinc ore body in anomaly package No. II occurs in the S_1_*W*^d^ limestone. Its extension direction is parallel to or nearly parallel to the strike of the strata, and its occurrence is basically the same as that of the surrounding rock (30–40° ∠ 60–70°). The ore bodies are closely related to the development of a ductile–brittle shear zone and have an outcrop width of 3–5 m and an outcrop length of about 100–450 m. The ore minerals are mainly galena and sphalerite, followed by chalcopyrite (Fig. 7a–c). The gangue minerals are calcite and quartz. The ore can be classified as carbonate–quartz vein type. The ore minerals are mainly endowed with mylonitized limestone, which has a xenomorphic granular texture and an infective, fine vein structure (Fig. 7d–f). The genetic type of this deposit, too, is hydrothermal sedimentary with structural alteration.

The field verification results for anomaly package No. I (①, ② and ③) show that the first-order hydroxyl anomaly points No. ① and No. ② correctly indicated the presence of a Cu–Pb–Zn ore belt. The Cu–Pb–Zn ore deposits are located in dense quartz veins. The remote sensing anomalies are distributed in beads along the quartz vein belt. The anomaly values are of relatively high magnitude and are in good agreement with the discovered ore bodies. No evidence of mineralization was found at the No. ③ secondary hydroxyl anomaly points, and only a small amount of silicification alteration was observed (Fig. 5). Field verification confirmed the presence of Pb–Zn–Cu deposits at each of the anomaly points of anomaly package No. II (④, ⑤ and ⑥). The ore body was mainly endowed with mylonitized limestone. The location of the ore body coincided well with the ASTER anomaly extracted (Fig. 5).

### Remote sensing geological characteristics and genesis of the copper–lead–zinc deposits

According to their metallogenic geological backgrounds, the Cu–Pb–Zn deposits in this area can be divided into mineralization belt No. I (corresponding to anomaly package No. I) and mineralization belt No. II (corresponding to anomaly package No. II). The ore-bearing rock series of mineralization belt No. I is phyllite interbedded with metasandstone, and it contains mainly copper deposits supplemented by lead–zinc deposits. The ore-bearing rock series of mineralization belt No. II is limestone, and it mainly consists of lead–zinc deposits supplemented by copper deposits.

The lithology of S_1_*W*^d^ is dark gray to gray-blue sericite phyllite, sericite silty sandy slate, medium and fine sandstone, limestone, carbonaceous phyllite, and interbedded carbonaceous–siliceous rock. It would have formed through hot water jet ore-bearing deposition in a rift environment, which would have provided a good metallogenic material basis for later mineralization. After the sedimentary period, under the action of bedding shear, the thin interbedded clastic rock assemblage would have developed poor rigidity and would have been easy to deform and metamorphose. A large number of metamorphic quartz veins with S1 foliation and weak mineralization precipitated. Later superimposition of a ductile–brittle fracture structure played a key role in the extraction, migration, enrichment, precipitation, and mineralization of mineral-bearing materials.

The later mineralization of the ductile and brittle fracture structures can be interpreted to have included the following: (1) Under the action of early brittle–ductile shear, the quartz veins formed during metamorphism broke down and ruptured, forming lenticular bodies; this was accompanied by penetration by an ore-bearing hydrothermal solution. However, the degree of mineralization was weak. (2) Under medium-term ductile–brittle shear, the mineral content in the tectonic hydrothermal fluid increased. Under the appropriate temperature and pressure conditions, enrichment caused mineral-bearing materials to precipitate; this was the main metallogenic period. An ore-bearing quartz–calcite hydrothermal solution was superimposed and enriched along fractures under the action of late-stage brittle fracture.

During the process of mineralization, differences in the affinity of the different elements cause copper to be polysilicon, while lead–zinc is polycarbonate. Therefore, copper–lead–zinc deposits occur mostly in quartz veins, while lead–zinc–copper deposits occur mostly in limestone.

### Remote sensing prospecting model for copper-lead–zinc deposits

Based on the foregoing analysis of the metallogenic geological characteristics of copper–lead–zinc deposits in the Heiqia area, the interpretation of the high-resolution remote sensing of ore-controlling elements, extraction of information on mineralization and alteration from remotely sensed anomalies, and field validation and correction, a comprehensive remote sensing prospecting model based on high-resolution remote sensing images was established (Table 1).

**Table 1:** The remote sensing geology prospecting model of copper–lead–zinc deposits in West Kunlun region.

Ore-controlling factors	Prospecting model
Geological conditions	
Geotectonic location	Northern margin of the Qiangtang–Tanggula Block, south of the Kangxiwa–Muzitage–Animaqing Late Paleozoic junction belt
Ore-hosting strata	D formation of the Early Silurian Wenquangou Group (S_1_*W*^d^)
Ore-bearing rock series	Copper deposits are closely related to quartz veins precipitated from phyllite intercalated with metasandstone, and lead–zinc deposits are closely related to limestone
Ore characteristics	The ore minerals are mainly chalcopyrite, galena, and sphalerite, gangue minerals are calcite and quartz, and the ore is an isomorphic–semi-automorphic granular aggregate. The ore structure is massive, infectious, and veined
Mineralization type	Silicification (quartz veins), limonitization, petrochemical, jarosite, etc
Ore-controlling structure	Under the action of bedding-parallel ductile–brittle shear, a large amount of ore-bearing tectonic hydrothermal precipitation–extraction–enrichment, and finally precipitation and mineralization
Genetic type of mineral deposit	Tectonic alteration types related to hot water basins
Remote sensing characteristics	
Remote sensing anomaly information	Hydroxyl abnormality
High-score image features	In the image generated by decorrelation analysis of the IKONOS 321 band combination, the copper mineralization zones exhibit interlaced gray-white, blue-gray, and blue tones with narrow strip-like patterns, while lead–zinc mineralization zones have gray-white, light gray-yellow, and yellowish-brown tones and strip-like patterns. The main alteration process associated with copper, lead, and zinc mineralization is silicification and a small amount of limonitization. Therefore, the main remotely sensed alteration anomalies are hydroxyl anomalies, with a small number of iron stain anomalies

Given the above discussion, we draw four conclusions as follows: (1) In the image generated by decorrelation analysis of the IKONOS 321 band combination, the copper ore of mineralization belt No. I has an interlaced gray-white, blue-gray, and blue tone and a narrow strip-like pattern, while the surrounding slate and phyllite lithology is dark blue, diabase and diorite rock bodies are red-brown, and the many distributed quartz veins are bright white. The lead–zinc ore of mineralization belt No. II has a gray-white, light gray-yellow, and yellow–brown tone and a belt-like shadow pattern, while the surrounding slate and phyllite are dark blue, diabase and diorite rock bodies are red-brown, and sandstone is light red. (2) The main alteration process associated with copper, lead, and zinc mineralization is silicification and a small amount of limonitization. Therefore, the main remotely sensed alteration anomalies are hydroxyl anomalies, with a small number of iron stain anomalies. (3) Six hydroxyl anomalies were delineated in the study area, of which five were found to be copper–lead–zinc deposits. The locations of the ore bodies coincide well with the ASTER anomalies extracted. (4) Two Cu–Pb–Zn mineralization belts are present in the study area. The ore-bearing rock series of mineralization belt No. I is phyllite interbedded with metasandstone, and the ore is mainly copper deposits supplemented by lead–zinc deposits. The ore-bearing rock series of mineralization belt No. II is limestone, and the ore is mainly lead–zinc deposits supplemented by copper deposits.

West Kunlun remains an area with the lowest degree of geological and mineral research along China's orogenic belt, due mainly to its cold and anoxic climate, strong topographic cutting, steep terrain, sparse population, and inconvenient transportation. Nonetheless, its scarce vegetation and bare bedrock make it suitable a target for the development of high-resolution remote sensing technology. Therefore, A remote sensing geological prospecting model for hot water basin-related structurally altered copper–lead–zinc deposits in the Wenquangou Group is established by us, providing a basis for future prospecting for similar minerals in the West Kunlun metallogenic belt.

## Methods

### Selection of remote sensing data

In this study, KONOS data was used to interpret strata, lithology and structure, and Aster data was used to extract alteration anomaly information of Cu–Pb–Zn deposits.

IKONOS satellite, manufactured by LOCKHEED MARTIN, could collect 1 m resolution full color and 4 m resolution multispectral images; the full color and multispectral images could be blended into the 1 m resolution color images. It can meet the needs of interpretation map for large-scale remote sensing geological survey of mineral resources. In this paper, the IKONOS remote sensing image data that was used was from June 2010.

ASTER is a multispectral imager mounted on the Terra satellite, whose received information includes the spectral reflectance of the ground in the VNIR (visible and near-infrared) and SWIR (thermal infrared) bands, and thermal radiation of the ground in the TIR range (thermal infrared) (Table 2)^57^. Due to the wide wavelength range, several bands, reasonable cost performance for the data, ASTER is widely used in extracting remote sensing (mineralization) alteration anomaly information^58–59^.

**Table 2:** The basic physics parameters of ASTER.

Subsystem	Sequence number of band	Wavelength range (nm)	spatial resolution (m)	Coverage area (km × km)
VNIR	1	520–600	15	60 × 60
	2	630–690		
	3N	780–860		
	3B	780–860		
SWIR	4	1,600–1,700	30	
	5	2,145–2,185		
	6	2,185–2,225		
	7	2,235–2,285		
	8	2,295–2,365		
	9	2,360–2,430		
TIR	10	8,125–8,475	90	
	11	8,475–8,825		
	12	8,925–9,275		
	13	10,250–10,950		
	14	10,950–11,650		

### Image enhancement processing

PCI, ENVI, and ERDAS remote sensing image processing software were used as image data processing platforms. A band-synthesis scheme was adopted to allow easy identification of geological structure and features, and ortho-correction, geometric precision correction, and fusion image processing procedures were applied. The processed images were used to produce a 1:50,000 standard remote sensing image map that adopts the 1980 Xi’an plane coordinate system, 1985 National elevation datum, and Gauss–Kruger projection.

According to the principle of band selection, the bigger the variance of band radiation and the smaller the band correlation is, the better. Through calculation of the best index, or optimum index factor for B3(R)B2(G)B1(B) and B4(R)B2(G)B1(B) of IKONOS, image fusion was performed using intensity–hue–saturation (IHS) transform, principal component transform, and PANSHARP. Afterward, a 1:50,000 topographic map and digital elevation model (DEM) data were used for selecting the rational polynomial coefficient parameter correction model to carry out orthorectification and cubic convolution resampling. Finally, the inlay mosaic method was used to create an image mosaic, and the base image for remote sensing interpretation was produced.

After the completion of image production, band combination transformation, decorrelation analysis, principal component analysis (PCA), differential stretching and shadow processing are used to enhance the image.

### Extraction of anomalies reflecting mineralization and alteration from ASTER Data

Several methods can be used to perform extraction of anomaly information in remote sensing (mineralization) alteration, including PCA, ratio analysis, and spectral angle analysis. The characteristic absorption spectra of various alteration anomalies and the corresponding relation between ASTER bands (Table 3) were considered to extract iron stain, aluminum hydroxyl (Al–OH), and magnesium hydroxyl (Mg–OH) anomalies by principal component transformation^60^.

**Table 3:** Abnormal absorption band of iron dye, hydroxyl.

Ion,perssad	Absorption spectrum/µm	ASTER band	Typical minerals
Fe^2+^, Fe^3+^	Fe^2+^: 1.1–2.4;	Band1, Band3	Pyrite, jarosite and magnetite
	Fe^3+^: 0.45, 0.55, 0.85, 0.90, 0.94		
AL–OH	AL–OH: 2.20	AL–OH: Band5, Band6	Kaolinite, Muscovite, serpentine

Thresholds were used to divide the anomalous areas by intensity, separate anomalous information layer by layer, and obtain a classification map of mineralization and alteration anomaly intensity. Threshold processing takes σ as the scale, with the mean + n (standard deviation) as the dynamic range of the principal component output. The threshold values of an iron stain anomaly (FCA) were 2.5σ for a first-order anomaly, 2σ for a second-order anomaly, and 1.5σ for a third-order anomaly. The threshold value of a hydroxyl anomaly was 3σ for a first-order anomaly, 2.5σ for a second-order anomaly, and 2σ for a third-order anomaly.

#### Anomaly separation

Filtering was applied to remove isolated anomalous pixels or to merge isolated pixels into adjacent anomalies to produce a continuous distribution. All remote sensing anomalies in the study area were screened according to spectral characteristics, anomalous characteristics, and metallogenic geological conditions. Spectral feature recognition, mutual validation of different types of anomalies, optimization of the spectral angle, geological interpretation, and other methods were used to remove the interference information twice, and a remote sensing anomaly map was obtained.

### Consent statement

All authors consent for publication of identifying information/images in an online open-access publication for this paper.
